# Understanding vestibular-related physiological functions could provide clues on adapting to a new gravitational environment

**DOI:** 10.1186/s12576-020-00744-3

**Published:** 2020-03-14

**Authors:** Hironobu Morita, Hiroshi Kaji, Yoichi Ueta, Chikara Abe

**Affiliations:** 1 Department of Physiology, Gifu University Graduate School of Medicine, Gifu, 501-1194 Japan; 2 Department of Physiology and Regenerative Medicine, Faculty of Medicine, Kindai University, Osakasayama, 589-8511 Japan; 3 Department of Physiology, School of Medicine, University of Occupational and Environmental Health, Kitakyushu, 807-8555 Japan

**Keywords:** Microgravity, Hypergravity, Vestibular system, Vestibulo-cardiovascular reflex, Muscle atrophy, Osteopenia, Gravity sickness, Hypophagia, Hypothermia

## Abstract

The peripheral vestibular organs are sensors for linear acceleration (gravity and head tilt) and rotation. Further, they regulate various body functions, including body stability, ocular movement, autonomic nerve activity, arterial pressure, body temperature, and muscle and bone metabolism. The gravitational environment influences these functions given the highly plastic responsiveness of the vestibular system. This review demonstrates that hypergravity or microgravity induces changes in vestibular-related physiological functions, including arterial pressure, muscle and bone metabolism, feeding behavior, and body temperature. Hopefully, this review contributes to understanding how human beings can adapt to a new gravitational environment, including the moon and Mars, in future.

## Background

The vestibular organ, which is located in the bony labyrinth of the inner ear, comprises two components; namely, the otoliths, which perceive linear acceleration and head tilt, and the semicircular canals, which perceive angular acceleration. The vestibular organ detects acceleration changes and converts them into neural signals, which are sent to the central nervous system to reflexively regulate physiological functions, including body stability (vestibulo-spinal reflex) [1], ocular movements (vestibulo-ocular reflex) [2, 3], sympathetic nerve activity (vestibulo-sympathetic reflex) [4–6], arterial pressure (vestibulo-cardiovascular reflex), food intake [7], and body temperature [5, 8–10]. There have been recent reports on vestibular-related muscle and bone metabolism [11–14] and increasing attention on the various vestibular system functions. The vestibular system is known to be highly plastic, i.e., its sensitivity might be altered upon exposure to a different gravitational environment. Given the aforementioned vestibular functions, plastic alteration of the vestibular system might be involved in spaceflight-associated (especially microgravity-associated) medical problems, including gravity sickness, balance disorder, orthostatic hypotension, muscle atrophy, and bone loss. Understanding the underlying mechanisms of these medical problems and establishing effective countermeasures is necessary for allowing space exploration requiring longer distances and time periods under a microgravity environment. In this review article, we focus on topics regarding vestibular-related physiological functions and their plastic alterations upon exposure to different gravitational environments, i.e., hypergravity and microgravity.

## Arterial pressure control and orthostatic hypotension

**Fig. 1:**
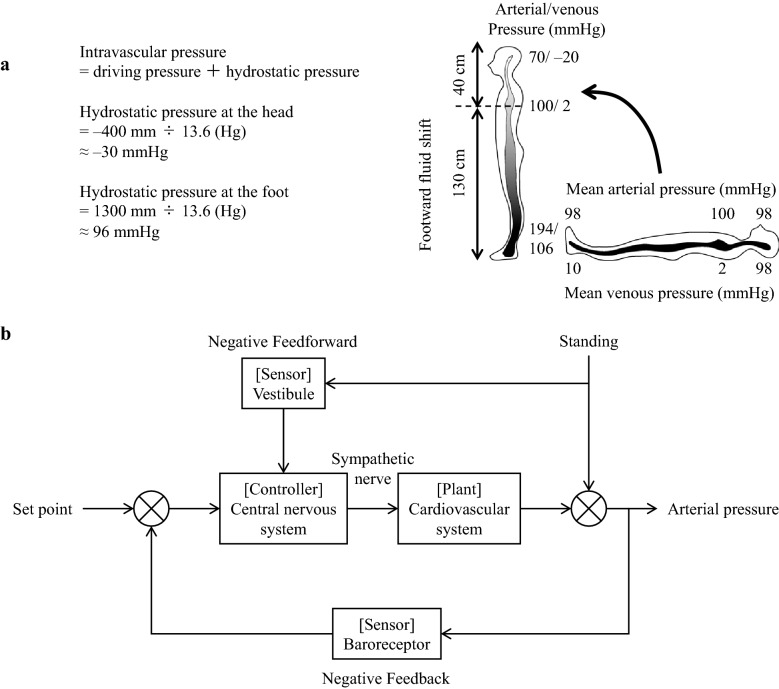
**a** Arterial and venous pressure change after supine-to-standing posture change. At the standing posture, the hydrostatic pressure of the foot increases to 96 mmHg while the venous pressure increases to 106 mmHg. Since the vein is more compliant, it dilates and 500 mL of blood is shifted to the lower body. **b** Block diagram of arterial pressure control at the standing posture. Revised Fig. 5 in reference 5

Gravity changes, i.e., in its magnitude or direction, are the most common and important disturbances to the cardiovascular system in beings living on the ground. These disturbances involve hydrostatic pressure changes that result from the gravity change. For example, a supine-to-standing posture change results in the longitudinal axis direction of the circulatory system and the gravity direction coinciding with each other. Consequently, the hydrostatic pressure of the lower body increases, the veins expand, and about 500 mL of blood accumulates in the lower body (Fig. 1a) [15]. Further, the blood volume in the thoracic cavity is reduced by 20% within 15 s of standing and there is a reduction in heart filling, cardiac output, and arterial pressure. Given the quick correction of these changes by the circulatory control mechanism, standing does not result in decreased arterial pressure in healthy individuals. However, more than 30% of older adults present orthostatic hypotension [16]. Further, 40% of astronauts returning from space have been reported to experience orthostatic hypotension [17]. The underlying mechanism has been reported to involve decreased circulating blood volume, baroreflex dysfunction, myocardial atrophy, etc. [18–21]. Further, recent reports have revealed an involvement of the vestibular system dysfunction [22, 23].

During voluntary rear-up behavior in rats, the arterial pressure is maintained by baroreflex and vestibulo-cardiovascular reflex [8]. Figure 1b presents a simple block diagram that represents the role of both reflexes [5]. The gravity directional change that accompanies the rear-up behavior induces a downward blood shift and a decrease in the arterial pressure. Simultaneously, this gravity change triggers the vestibular system and reflexively increases the sympathetic activity and arterial pressure (vestibulo–cardiovascular reflex) [4, 5]. This reflex is a type of feedforward control that quickly operates before the arterial pressure changes due to the blood shift upon gravity directional changes, and subsequently prevents falling of the arterial pressure. However, since the adjustment is based on gravity change rather than arterial pressure change, a control error occurs. The baroreceptor reflex corrects this control error, which is a feedback control system. Therefore, the vestibular system and baroreceptor reflexes maintain arterial pressure in a cooperative and interactive manner [5, 8].

Animal experiments allow examination of the role of the vestibular system in arterial pressure control by comparing the arterial pressure response to postural changes in animals with an intact vestibular system to that in animals with a lesioned vestibular system. However, it is impossible to use invasive and irreversible methods, such as vestibular lesion (VL), in humans. Therefore, there is a need for a non-invasive and reversible vestibular blocking method that can be used in place of VL. An effective alternative is galvanic vestibular stimulation (GVS), which stimulates vestibular afferents using surface electrodes placed on the bilateral mastoid processes of the temporal bone. Animal studies have reported that GVS attenuates gravity change-induced vestibulo-cardiovascular reflex similarly to VL [24]; therefore, it confirmed its effectiveness. This method has been used to confirm that the magnitude of the vestibulo-cardiovascular reflex at 60º head-up tilt is about 15 mmHg [9, 23]. Further, a study has reported a correlation between the degree of arterial pressure fall at the onset of 60º head-up tilt and the deterioration of otolith function as estimated by a subjective visual vertical study [25].

The vestibular system is known to be highly plastic with its function changing under different gravitational environments. We have previously reported a reduction in the sensitivity of vestibulo-cardiovascular reflex in rats reared in hypergravity environments [26–29]. This decrease is not due to the excessive gravity itself; rather, it is considered to result from use-dependent plasticity caused suppression of daily activities in the hypergravity environment and reduction of daily input to the vestibular system. In a 1*g* environment, rats rear up approximately 400–500 times a day; however, in a hypergravity environment, rats rear up only a few times a day. Further, their head movements, which indicate input to the vestibular system, are 10–20% of those under a 1*g* environment [28, 29]. Moreover, rats reared under restricted behavior in a narrow cage present a similar suppression of vestibulo-cardiovascular reflex that is comparable to that in a hypergravity environment [29].

Similar findings have been reported by human studies. In elderly people with reduced daily physical activity, the arterial pressure drops by about 20 mmHg upon 60º head-up tilt regardless of the presence or absence of GVS [9]. This indicates that an almost lack of ability of elderly individuals to regulate the vestibulo-cardiovascular reflex; further, reduced vestibular function is involved in the orthostatic hypotension often seen in elderly people.

**Fig. 2:**
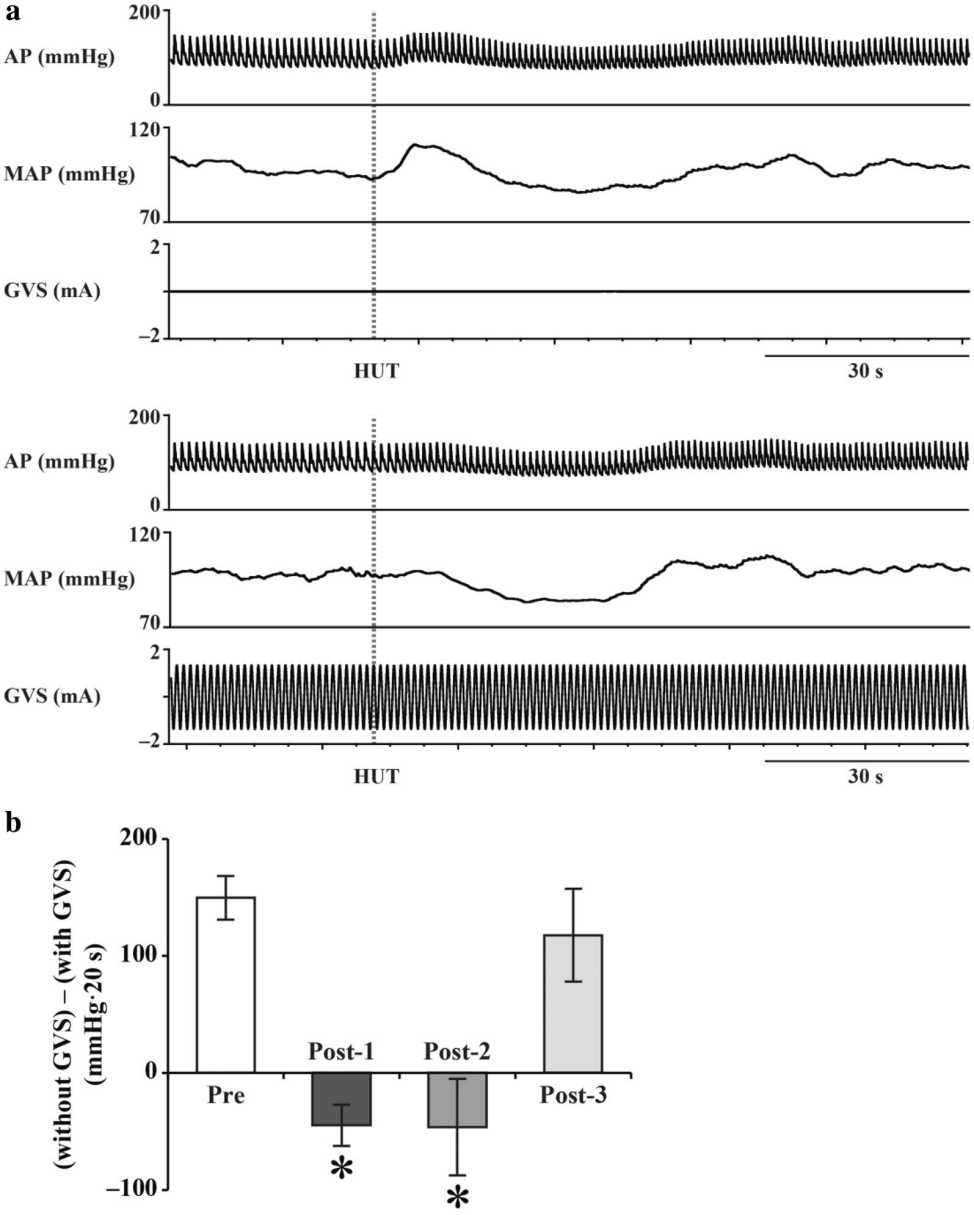
**a** Arterial pressure (AP) and mean AP (MAP) responses to 60º head-up tilt (HUT) with (lower panel) and without (upper panel) galvanic vestibular stimulation (GVS). The magnitude of vestibulo-cardiovascular reflex can be estimated by the AP response difference between that without and that with GVS. **b** Sum of differences in Δ AP between the initial response (within the first 20 s) to HUT without and with GVS [(without GVS) − (with GVS)], at Pre (2–4 months before launch), Post-1 (1–4 days after return), Post-2 (11–15 days after return), and Post-3 (2 months ± 12 days after return). Data are shown as mean ± standard error of the mean for six participants. *P < 0.05 vs. Pre. Revised Figs. 1 and 3 in reference 23.

In a microgravity environment, rotational acceleration and linear acceleration are maintained; however, there is a loss of input to the otolith due to head tilt. Specifically, input to the semicircular canal is maintained, but input to the otolith is reduced. Under these circumstances, there might be use-dependent plastic alterations to the vestibular otolith system with a reduction of the ability to adjust the vestibulo-cardiovascular reflex. A reduction in the ocular counter-rolling response, which is an otolith-driven reflex, has been reported upon return from long-term spaceflight [3]. Recently, Hallgren et al. reported a significant correlation between decreased otolith function and reduced arterial pressure response upon head-up tilt upon returning from spaceflight, which suggests that a deconditioned otolith system causes orthostatic intolerance [22]. Specifically, we previously estimated the magnitude of vestibulo-cardiovascular reflex upon return from spaceflight and found that it was non-operational and that it gradually recovered over the next 2 months [23] (Fig. 2).

Further, we have previously reported that GVS prevents hypergravity-induced plastic alterations of the vestibulo-cardiovascular reflex [28]. This could be due to GVS complementing the hypergravity-induced reduction in the phasic vestibular input. If the same occurs in microgravity, then incorporating GVS with training in the International Space Station might be a new countermeasure against vestibular deterioration.

## Muscles and bones

Microgravity is well known to induce muscle atrophy and bone loss in astronauts [30–32]. A previous study suggested that microgravity-induced muscle wasting and osteopenia are partly due to enhanced bone resorption and reduced calcium absorption in the intestines [4]; however, the effects of gravity change on osteoblastic bone formation and osteoclastic bone resorption remain unclear [33]. A recent study reported that the microgravity-induced effect on syncytin-A expression stimulated osteoclast formation independently of receptor activation of nuclear factor-kappa B ligand (RANKL), a crucial bone resorption factor, in mouse monocytic RAW264.7 cells [34]. Regarding osteoblasts, microgravity suppresses cell metabolism by impairing the cell mitochondrial energy state in human osteoblasts; further, it represses rat osteoblast differentiation by affecting primary cilia, which partly sense mechanical stress [35, 36]. Moreover, spaceflight has been reported to result in enhanced bone resorption by inducing osteocyte death and the subsequent bone mass deterioration and microstructure in mice [37].

Further, gravitational unloading induces muscle atrophy and decreases the contraction capacity of anti-gravity muscles in rodents and humans [9]. Hypergravity enhances the mass of anti-gravity muscles and inhibits ovariectomy-induced osteopenia in rodents [38, 39]. Tominari et al. recently reported that hypergravity and microgravity have contrasting effects on bone and muscle in mice [40], which suggests that animal models kept in hypergravity environment could be used to investigate the microgravity influences on muscles and bones.

Gravity change induces plastic alteration of the vestibular system that links the motor and sympathetic nervous systems, which are crucial muscle and bone regulators. Several studies have reported that labyrinthectomy or VL reduces bone mass with partial involvement in the sympathetic nervous system in rodents, which indicates that the vestibular system regulates bone metabolism [11, 12, 41]. Regarding the vestibular system role in skeletal muscles, labyrinthectomy induces changes in muscle fiber morphology and function in ferrets. Further, the vestibular system modulates muscle fiber size and transcription factor expression in rats [14, 42]. Clinical studies have reported a relation between benign paroxysmal positional vertigo with the vestibular dysfunction and osteoporosis, high bone turnover, and vitamin D deficiency in patients with osteoporosis [43–45]. Taken together, these findings indicate that vestibular system regulates both skeletal muscles and bones.

There have been reports indicating muscle–bone interaction based on the clinical relationships between sarcopenia and osteoporosis, as well as the common effects of numerous factors, including genetic, mechanical stress, endocrine factors, nutrition, and inflammation, on both skeletal muscles and bones [46–48]. In astronauts, microgravity-induced muscle wasting recovers faster than osteopenia [49]. Therefore, we speculated that gravity change influences muscle–bone interactions by affecting muscle-derived factors (myokines) that link muscle to bone, which might have a partial involvement in microgravity-induced changes in muscle and bones. We investigated the hypergravity effects on muscles and bones in mice using the gondola-type centrifuge in a 3*g* environment with or without VL [13]. We found that a 4-week exposure to hypergravity in a 3*g* environment increased the anti-gravity muscle weight and fiber size, as well as the expression of muscle differentiation genes, including MyoD. Moreover, it increased the body weight-adjusted trabecular bone mass. VL and inhibition of the sympathetic nervous system using an adrenergic *β* blocker, propranolol, antagonized the hypergravity-induced muscle and bone changes. These findings suggest that gravity change affects muscles and bones through the vestibular and subsequent sympathetic outflow in mice [49]. We performed similar experiments in mice using hypergravity in a 2*g* environment for 2 weeks [50]. We found that it enhanced osteoblast differentiation partly through the vestibular system, which suggests that the vestibular system might contribute to the adaptive response of bone tissues during gravity change [50]. However, the effects of hypergravity, such as 2 or 3*g*, on bone and muscle mass are still currently unclear [13, 40, 50].

**Fig. 3:**
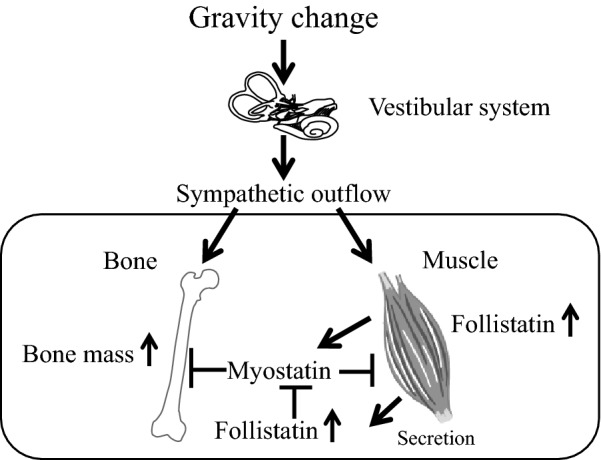
Role of follistatin in the effects of gravity change on muscle and bone. Follistatin suppresses the action of myostatin, an inhibitor of skeletal muscle mass and a stimulator of bone resorption. Hypergravity enhances follistatin expression in skeletal muscles through the vestibular system in mice. Circulating follistatin induced by gravity change might be involved in hypergravity-enhanced bone mass by inhibiting myostatin-induced bone resorption

Comprehensive DNA microarray analysis of mouse anti-gravity muscle samples indicated that the gene for FK506 binding protein 5 (FKBP5) is responsible for the hypergravity-induced muscle mass increase through the vestibular system in mice [51]. Numerous myokines secreted from skeletal muscles have positive or negative effects on bones [47] and might be involved in the effects of gravity changes on muscles and bones through the vestibular system. Although follistatin is a known antagonist of myostatin, a crucial myokine that links muscle to bone, hypergravity enhances follistatin expression in anti-gravity muscle and its subsequent secretion to the bloodstream through the vestibular system in mice. Further, there seems to be an association between follistatin and hypergravity-induced bone mass increase (Fig. 3) [52]. Taken together, these findings indicate that vestibular system plasticity might be crucial for the microgravity-induced effects on muscles and bones through various factors, including follistatin and FKBP5. However, the factors through which gravity change and mechanical stress modulate muscles and bones seem to be slightly different [53]. Further studies are required for detailed clarification of the underlying mechanisms of microgravity-induced effects on muscles and bones. Controlling the vestibular system and clarifying the critical factors that maintain muscles and bones in response to gravity change might be useful to prevent microgravity- or immobilization-induced muscle wasting and osteopenia.

## Gravity sickness and hypophagia

Approximately 60–80% of astronauts suffer from space motion sickness during the first 2–3 days in microgravity [54]. The more appropriate term for it would be gravity sickness because symptoms similar to space motion sickness occur immediately after returning to the ground or being exposed to hypergravity. These symptoms, which affect the performance of astronauts, are important problems that should be solved. Responses in different gravity environments indicate certain biological changes. Transient hypophagia and subsequent weight loss are common biological responses to centrifugation-induced hypergravity [7, 55, 56]. Feeding is controlled by the central nervous system via various neuropeptide-containing neurons of the hypothalamus [7, 57, 58]. It has been reported that there are neural inputs from the vestibular system to the hypothalamus [59]. Morita et al. reported, in mice, a marked increase in fos immunoreactivity in the paraventricular nucleus (PVN) after a 90-min exposure to 2*g* hypergravity, which was abolished by VL [56]. Since the reduced food intake is fully or partially ameliorated by the VL, the vestibular system is also, at least partially, involved in the hypergravity-induced hypophagia [7]. However, it was unveiled how gene expression of hypothalamic feeding-regulating neuropeptides is altered in hypergravity-induced hypophagia and whether vestibular function is involved in the modulation of neuropeptide expression in the hypergravity environment.

We examined the gene expression of hypothalamic feeding-regulating neuropeptides at 3 days, 2 weeks, and 8 weeks after exposure to a 2*g* environment induced using centrifugation and compared the expression levels of various neuropeptides between sham-operated (Sham) or VL mice. Three anorexigenic neuropeptides [corticotrophin-releasing hormone (CRH), pro-opiomelanocortin (POMC), and cocaine- and amphetamine-regulated transcript (CART)] and four orexigenic neuropeptides [neuropeptide Y (NPY), agouti-related protein (AgRP), melanin-concentrating hormone (MCH), and orexin] were studied using in situ hybridization histochemistry.

The summary of the results is presented in Table 1 [60]. After a 3-day exposure to hypergravity, the gene expression of CRH in the PVN was altered only in Sham-*2g* mice but not in VL-2*g* mice. CRH is known to be involved in the stress response and suppression of feeding behavior [57, 61, 62]. Thus, it is suggested that the increased gene expression of CRH is a result of the stress response induced by the hypergravity environment. A previous study reported that in rats, after a 90-min exposure to 2*g*, the immunoreactivity of CRH in the PVN significantly increased, but it was attenuated by VL [63]. Thus, CRH-producing neurons in the PVN may receive neuronal input from the vestibular system.

**Table 1:** Summary of change in the gene expression of the hypothalamic feeding- regulating neuropeptides after exposure to 2*g* hypergravity compared with sham-1*g*

mRNA	After 3 days exposure to hypergravity		After 2 weeks exposure to hypergravity		After 8 weeks exposure to hypergravity	
	sham-2*g*	VL-2*g*	sham-2*g*	VL-2*g*	sham-2*g*	VL-2*g*
*CRH*	↑	→	↑	→	→	→
*POMC*	↓	↓	↑	→	→	→
*CART*	↓	↓	→	→	→	→
*NPY*	↑	↑	→	→	→	→
*AgRP*	↑	↑	→	→	→	→
*MCH*	→	→	→	→	→	→
*orexin*	↑	↑	→	→	→	→

↑ up regulation;

→ no significant change

↓down regulation

We believe that the changes in gene expression of the hypothalamic feeding-regulating neuropeptides have different implications at each time point. Starved state induced both upregulation in NPY/AgRP and orexin neurons and downregulation in the POMC/CART neuron [58, 64, 65]. Acute hypophagia-induced starvation after a 3-day exposure to hypergravity may cause the downregulation of anorexigenic neuropeptides such as POMC and CART and upregulation of orexigenic neuropeptides such as NPY, AgRP, and orexin; whereas, after a 2-week exposure to hypergravity, gene expression of the CRH and POMC showed a dramatic increase in Sham-2*g* mice but not in VL-2*g* mice. These changes may be due to inadequate adaptation to the different gravity rather than starvation or fasting, since after a 2 weeks of hypergravity exposure, food consumption had recovered [7]. Thus, we speculate that it takes more than 2 weeks for the vestibular system to adapt to the different gravity. The gene expression of hypothalamic feeding-regulating neuropeptides was not different after an 8-week exposure to hypergravity. It was considered that the vestibular system adapts to the different gravity in 8 weeks, as the vestibular system is known to be highly plastic and able to adapt to novel gravitational environments [26, 56].

## Gravity sickness and hypothermia

Gravitational change is a stressor that is detected by the peripheral vestibular apparatus, including otolith organs. Short-term gravity changes, including microgravity and hypergravity, or GVS activate the sympathetic nervous system in rodents and humans [24, 26, 66, 67]. Therefore, it is possible that stress detected by the peripheral vestibular organs induces hyperthermia with a similar response to that of other stressors, including air jet, restraint, social defeat, novel cage, cage switch, and handling [68]. However, gravitational change, especially long-term hypergravity loading, has been reported to induce hypothermia rather than hyperthermia [10, 69, 70].

Long-term and long-lasting inescapable stress induces hypothermia [71]. Notably, there is a body temperature decrease in animals subjected to restraint or immobilization stress. Further, other stress forms, including food deprivation [72], hypoxia [73], and rotation [74], lead to torpor and decreased oxygen consumption. Hypergravity-induced hypothermia could result from two synergistic effects; namely, increased heat dissipation and reduced thermogenesis. Vasomotor control of the rodent hairless tail is crucial for the dissipation of excess body heat [75]. For example, the blood flow in rat tails increases 3- to 4-fold when the ambient temperature exceeds a threshold level of approximately 27 °C [76]. Rotation and shaking stimulation have been previously reported to increase the tail temperature in rats and house musk shrew (*Suncus murinus*) [77]. This indicates that increased heat dissipation from the tail occurs during the stimulation of the peripheral vestibular organs. However, tailless rats did not show attenuation of hypergravity load-induced hypothermia, which suggests that heat loss from the tail is not involved in the response [69].

Regarding heat production, brown adipose tissue (BAT) activation is a major cause of thermogenesis in rodents. The BAT mass in humans is approximately 10% that in mice; however, recent studies indicate that it plays a thermogenic role [78]. The stimulation of the peripheral vestibular organs seems to influence BAT activity; specifically, provocative motion reduces BAT thermogenesis in musk shrews [77]. A hypergravity study using rats reported that oxygen consumption, which was higher during cold stimulation, decreased by 50% during the loading [70], which might induce decreased BAT thermogenesis. Therefore, hypergravity suppresses heat production through the BAT, which might subsequently lead to hypothermia.

BAT sympathetic nerve activity has been reported to show a decrease dependent on the increase in the electrical stimulation frequency to the vagal afferents [79]. In the central nervous system, the signal is transmitted via the vagal afferents to the nucleus of the solitary tract. Subsequently, it is transmitted to the rostral raphe pallidus area, which contains BAT sympathetic premotor neurons. This pathway is thought to be involved in hypothermia induced by vagal afferents stimulation. Electrical-stimulated vagal afferent-induced inhibition of BAT sympathetic nerve activity has been reported to be prevented by blocking ionotropic glutamate receptors in the termination side of the vagal afferents in the nucleus of the solitary tract, as well as by nanoinjection of GABAA receptor antagonists in the rostral raphe pallidus [79]. Contrastingly, there are no direct projections from the vestibular nuclear complex to the rostral raphe pallidus, magnus, or obscurus [80]. However, there is a neural projection from the vestibular nuclear complex to the nucleus of the solitary tract [81, 82]. Taken together, these findings suggested that vestibular input-induced neural activity inhibition in the rostral raphe pallidus area via the nucleus of the solitary tract might be involved in hypergravity-induced hypothermia.

Previous studies have reported a relationship between gravity sickness and reduced body core temperature. The vestibular system might be involved in gravity sickness-induced hypothermia since otoconia deletion using global knockdown mice (NADPH oxidase 3 mutation) was reported to suppress hypergravity-induced hypothermia [10]. In humans, participants experiencing nausea during caloric ear stimulation exhibited increased tonic skin conductance in the fingers and an increased sweating rate on the forehead [83].

**Fig. 4:**
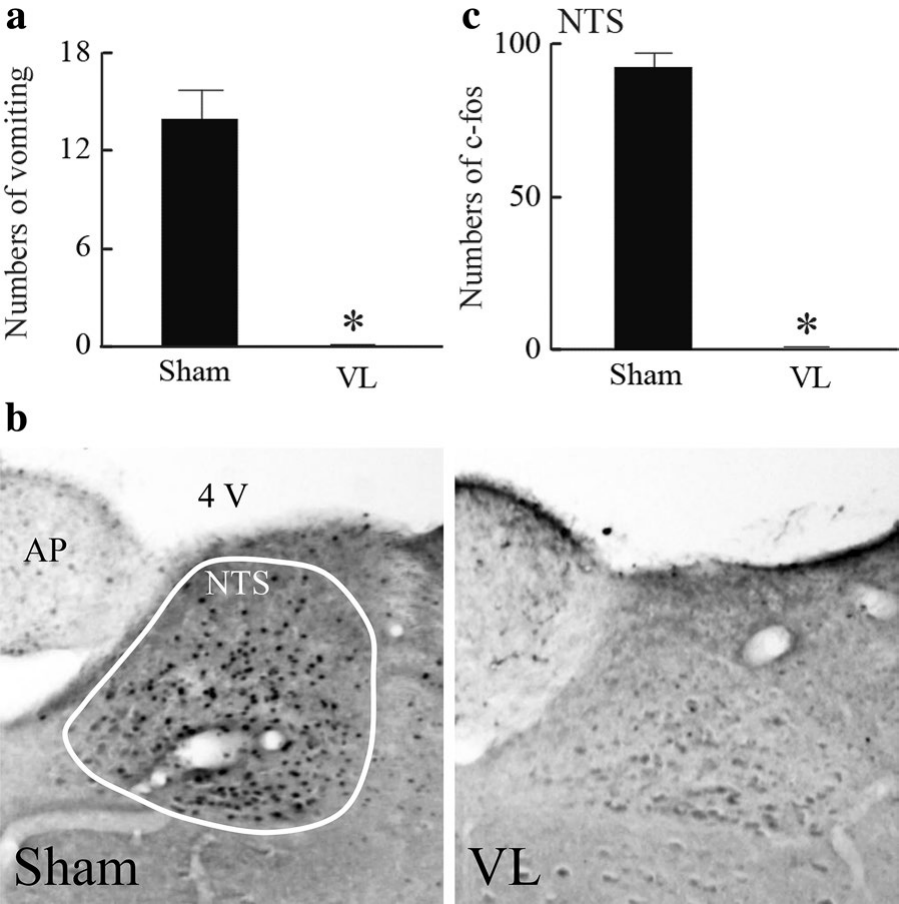
**a** Numbers of vomiting episodes during 10-min 2*g* exposure in a musk shrew with (sham) or without (VL) peripheral vestibular apparatus. **b** Representative images of fos-expressing cells in the nucleus of the solitary tract (NTS). **c **Summarized data for the number of fos-expressing cells in the NTS. Data are shown as mean ± standard error of the mean. *P < 0.05 vs. Sham. Revised Figs.1, 2, and 3 in reference 67.

In contrast to humans, it is difficult to determine whether rodents experience hypergravity-induced gravity sickness since they do not show an emetic response. In musk shrews, which are good models for gravity sickness since they show emesis, it has been reported that the number of vomiting episodes was 14 ± 2 during a 10-min 2*g* exposure. This was accompanied by intense fos expression in the medial vestibular nucleus, nucleus of the solitary tract, area postrema, and PVN. This was completely reversed by VL [84] (Fig. 4). Allotriophagy, which is a motion sickness index, has been observed in rats during hypergravity load [85, 86]. Drugs for motion sickness appear to be effective. Rotation-induced hypothermia, which involves stimulation of the semicircular canals rather than the otolith organs, has been reported to be suppressed by 5-HT3 receptor blockade [77]. Additionally, some studies have suggested the involvement of the vestibular efferents, which terminate on the vestibular hair cells and release acetylcholine. Shaking-induced hypothermia is suppressed in mice lacking the α9 cholinoreceptor subunit, which is predominantly expressed in the vestibular hair cells [74]. In humans, rotation with enhanced head movements, which facilitates motion sickness, was reported to result in decreased body temperature compared to that after rotation only [87].

Although not all observed changes have been reported for all species, gravity sickness-induced hypothermia appears to be quite common and has been reported in mice, musk shrews, and rats. The reason for and physiological significance of this hypothermic effect remains unclear. Unfortunately, there is no evidence regarding an evolutionary advantage resulting from the appearance of this response. In a natural evolution, it is difficult to imagine how animals, except for human beings, could be subjected to rotational or linear provocative motion. Therefore, it can be assumed that gravity sickness-induced hypothermia is not a product of evolutionary pressure; specifically, gravity sickness might be a disturbance created by human technology development. With the exclusion of pharmacologically and cold-induced hypothermia, other situations of hypothermia occurring in response to environmental stressors are toxic and/or septic shock [88]. A common feature between gravity sickness and toxic shock is nausea presence, which is a defense mechanism against intoxication. Rat experiments have shown that hypothermia and cold-seeking behavior upon toxic shock is not only defensive, but also actually critical for survival [89, 90] with the adaptive reaction being to reduce the tissue demands for oxygen, which is critical for survival during intoxication [91]. Therefore, one could speculate that since both nausea and hypothermia develop during gravity sickness, they could involve activation of the same defense mechanism and might be a result of natural selection to survive. Further, gravity sickness can be considered an adaptive response evoked by an inappropriate stimulus.

## Conclusion

**Fig. 5:**
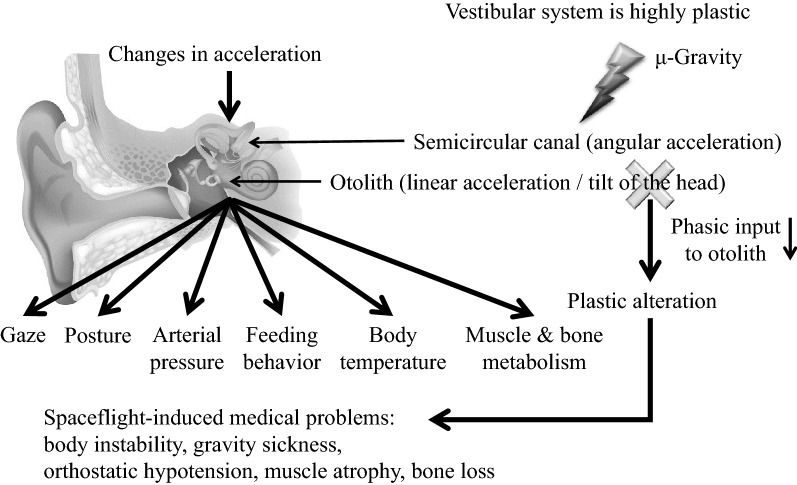
Summary of this review

Figure 5 presents a summary of the conclusion of this review. The vestibular system controls various physical functions, including body stability, sympathetic nerve activity, arterial pressure, feeding behavior, body temperature, and muscle and bone metabolism. However, it is highly plastic and its function is altered upon exposure to different gravitational environments. Changes in vestibular-related physical control functions might involve use-dependent plasticity due to decreased phasic input to the otolith organ. Further, vestibular dysfunction can be ameliorated through appropriate stimulation of the vestibular system.

## Data Availability

Data sharing is not applicable to this article as no datasets were generated or analyzed during the current study.

## Abbreviations

VL: Vestibular lesion
GVS: Galvanic vestibular stimulation
PVN: Paraventricular nucleus
CRH: Corticotrophin-releasing hormone
POMC: Pro-opiomelanocortin
CART: Cocaine- and amphetamine-regulated transcript
NYP: Neuropeptide Y
AgRP: Agouti-related protein
MCH: Melanin-concentrating hormone
BAT: Brown adipose tissue

## References

[1] Reschke MF, Bloomberg JJ, Harm DL, Paloski WH, Layne C, McDonald V (1998). Posture, locomotion, spatial orientation, and motion sickness as a function of space flight. Brain Res Rev.

[2] Clarke AH (1998). Vestibulo-oculomotor research and measurement technology for the space station era. Brain Res Rev.

[3] Hallgren E, Kornilova L, Fransen E, Glukhikh D, Moore ST, Clement G, Van Ombergen A, MacDougall H, Naumov I, Wuyts FL (2016). Decreased otolith-mediated vestibular response in 25 astronauts induced by long-duration spaceflight. J Neurophysiol.

[4] Yates BJ, Bolton PS, Macefield VG (2014). Vestibulo-sympathetic responses. Comp Physiol.

[5] Gotoh TM, Fujiki N, Matsuda T, Gao S, Morita H (2004). Roles of baroreflex and vestibulosympathetic reflex in controlling arterial blood pressure during gravitational stress in conscious rats. Am J Physiol Regul Integr Comp Physiol.

[6] Ray CA (2000). Interaction of the vestibular system and baroreflexes on sympathetic nerve activity in humans. Am J Physiol Heart Circ Physiol.

[7] Abe C, Tanaka K, Iwata C, Morita H (2010). Vestibular-mediated increase in central serotonin plays an important role in hypergravity-induced hypophagia in rats. J Appl Physiol.

[8] Abe C, Kawada T, Sugimachi M, Morita H (2011). Interaction between vestibulo-cardiovascular reflex and arterial baroreflex during postural change in rats. J Appl Physiol.

[9] Tanaka K, Abe C, Awazu C, Morita H (2009). Vestibular system plays a significant role in arterial pressure control during head-up tilt in young subjects. Auton Neurosci.

[10] Fuller PM, Jones TA, Jones SM, Fuller CA (2002). Neurovestibular modulation of circadian and homeostatic regulation: vestibulohypothalamic connection?. Proc Natl Acad Sci USA.

[11] Vignaux G, Besnard S, Ndong J, Philoxene B, Denise P, Elefteriou F (2013). Bone remodeling is regulated by inner ear vestibular signals. J Bone Miner Res.

[12] Vignaux G, Ndong JD, Perrien DS, Elefteriou F (2015). Inner ear vestibular signals regulate bone remodeling via the sympathetic nervous system. J Bone Miner Res.

[13] Kawao N, Morita H, Obata K, Tamura Y, Okumoto K, Kaji H (2016). The vestibular system is critical for the changes in muscle and bone induced by hypergravity in mice. Physiol Rep..

[14] Luxa N, Salanova M, Schiffl G, Gutsmann M, Besnard S, Denise P, Clarke A, Blottner D (2013). Increased myofiber remodelling and NFATc1-myonuclear translocation in rat postural skeletal muscle after experimental vestibular deafferentation. J Vestib Res.

[15] Robertson D (2008). The pathophysiology and diagnosis of orthostatic hypotension. Clin Auton Res.

[16] Salminen M, Raiha I, Heinonen J, Kivela SL (2012). Morbidity in aged Finns: a systematic review. Arch Gerontol Geriatr.

[17] Meck JV, Waters WW, Ziegler MG, deBlock HF, Mills PJ, Robertson D, Huang PL (2004). Mechanisms of postspaceflight orthostatic hypotension: low alpha1-adrenergic receptor responses before flight and central autonomic dysregulation postflight. Am J Physiol Heart Circ Physiol.

[18] Fritsch-Yelle JM, Charles JB, Jones MM, Beightol LA (1985). Eckberg DL (1994) Spaceflight alters autonomic regulation of arterial pressure in humans. J Appl Physiol.

[19] Meck JV, Reyes CJ, Perez SA, Goldberger AL, Ziegler MG (2001). Marked exacerbation of orthostatic intolerance after long- vs. short-duration spaceflight in veteran astronauts. Psychosom Med.

[20] Perhonen MA, Franco F, Lane LD, Buckey JC, Blomqvist CG, Zerwekh JE, Peshock RM, Weatherall PT, Levine BD (2001). Cardiac atrophy after bed rest and spaceflight. J Appl Physiol.

[21] Convertino VA (2002). Mechanisms of microgravity induced orthostatic intolerance: implications for effective countermeasures. J Gravit Physiol.

[22] Hallgren E, Migeotte PF, Kornilova L, Deliere Q, Fransen E, Glukhikh D, Moore ST, Clement G, Diedrich A, MacDougall H, Wuyts FL (2015). Dysfunctional vestibular system causes a blood pressure drop in astronauts returning from space. Sci Rep.

[23] Morita H, Abe C, Tanaka K (2016). Long-term exposure to microgravity impairs vestibulo-cardiovascular reflex. Sci Rep.

[24] Abe C, Tanaka K, Awazu C, Morita H (2008). Strong galvanic vestibular stimulation obscures arterial pressure response to gravitational change in conscious rats. J Appl Physiol.

[25] Tanaka K, Abe C, Sakaida Y, Aoki M, Iwata C, Morita H (2012). Subsensory galvanic vestibular stimulation augments arterial pressure control upon head-up tilt in human subjects. Auton Neurosci.

[26] Abe C, Tanaka K, Awazu C, Chen H, Morita H (2007). Plastic alteration of vestibulo-cardiovascular reflex induced by 2 weeks of 3-G load in conscious rats. Exp Brain Res.

[27] Morita H, Abe C, Awazu C, Tanaka K (2007). Long-term hypergravity induces plastic alterations in vestibulo-cardiovascular reflex in conscious rats. Neurosci Lett.

[28] Abe C, Tanaka K, Awazu C (1985). Morita H (2009) Galvanic vestibular stimulation counteracts hypergravity-induced plastic alteration of vestibulo-cardiovascular reflex in rats. J Appl Physiol.

[29] Abe C, Shibata A, Iwata C, Morita H (2010). Restriction of rear-up-behavior-induced attenuation of vestibulo-cardiovascular reflex in rats. Neurosci Lett.

[30] Bloomfield SA, Martinez DA, Boudreaux RD, Mantri AV (2016). Microgravity stress: bone and connective tissue. Compr Physiol.

[31] Orwoll ES, Adler RA, Amin S, Binkley N, Lewiecki EM, Petak SM, Shapses SA, Sinaki M, Watts NB, Sibonga JD (2013). Skeletal health in long-duration astronauts: nature, assessment, and management recommendations from the NASA Bone Summit. J Bone Miner Res.

[32] Smith SM, Zwart SR, Heer M, Hudson EK, Shackelford L, Morgan JL (2014). Men and women in space: bone loss and kidney stone risk after long-duration spaceflight. J Bone Miner Res.

[33] Smith SM, Wastney ME, O'Brien KO, Morukov BV, Larina IM, Abrams SA, Davis-Street JE, Oganov V, Shackelford LC (2005). Bone markers, calcium metabolism, and calcium kinetics during extended-duration space flight on the mir space station. J Bone Miner Res.

[34] Ethiraj P, Link JR, Sinkway JM, Brown GD, Parler WA, Reddy SV (2018). Microgravity modulation of syncytin-a expression enhance osteoclast formation. J Cell Biochem.

[35] Michaletti A, Gioia M, Tarantino U, Zolla L (2017). Effects of microgravity on osteoblast mitochondria: a proteomic and metabolomics profile. Sci Rep.

[36] Shi W, Xie Y, He J, Zhou J, Gao Y, Wei W, Ding N, Ma H, Xian CJ, Chen K, Wang J (2017). Microgravity induces inhibition of osteoblastic differentiation and mineralization through abrogating primary cilia. Sci Rep.

[37] Gerbaix M, Gnyubkin V, Farlay D, Olivier C, Ammann P, Courbon G, Laroche N, Genthial R, Follet H, Peyrin F, Shenkman B, Gauquelin-Koch G, Vico L (2017). One-month spaceflight compromises the bone microstructure, tissue-level mechanical properties, osteocyte survival and lacunae volume in mature mice skeletons. Sci Rep.

[38] Frey M, von Kanel-Christen R, Stalder-Navarro V, Duke PJ, Weibel ER, Hoppeler H (1997). Effects of long-term hypergravity on muscle, heart and lung structure of mice. J Comp Physiol B.

[39] Ikawa T, Kawaguchi A, Okabe T, Ninomiya T, Nakamichi Y, Nakamura M, Uehara S, Nakamura H, Udagawa N, Takahashi N, Nakamura H, Wakitani S (2011). Hypergravity suppresses bone resorption in ovariectomized rats. Adv Space Res.

[40] Tominari T, Ichimaru R, Taniguchi K, Yumoto A, Shirakawa M, Matsumoto C, Watanabe K, Hirata M, Itoh Y, Shiba D, Miyaura C, Inada M (2019). Hypergravity and microgravity exhibited reversal effects on the bone and muscle mass in mice. Sci Rep.

[41] Levasseur R, Sabatier JP, Etard O, Denise P, Reber A (2004). Labyrinthectomy decreases bone mineral density in the femoral metaphysis in rats. J Vestib Res.

[42] Shall MS, Lanzino DJ, Van Cleave S, Goldberg SJ (2005). Neonatal bilabyrinthectomy leads to changes in skeletal muscle fiber form and function. J Vestib Res.

[43] Bigelow RT, Semenov YR, Anson E, du Lac S, Ferrucci L, Agrawal Y (2016). Impaired vestibular function and low bone mineral density: data from the baltimore longitudinal study of aging. J Assoc Res Otolaryngol.

[44] Jeong SH, Choi SH, Kim JY, Koo JW, Kim HJ, Kim JS (2009). Osteopenia and osteoporosis in idiopathic benign positional vertigo. Neurology.

[45] Lee SB, Lee CH, Kim YJ, Kim HM (2017). Biochemical markers of bone turnover in benign paroxysmal positional vertigo. PLoS ONE.

[46] Kaji H (2013). Linkage between muscle and bone: common catabolic signals resulting in osteoporosis and sarcopenia. Curr Opin Clin Nutr Metab Care.

[47] Kaji H (2016). Effects of myokines on bone. Bonekey Rep.

[48] Kawao N, Kaji H (2015). Interactions between muscle tissues and bone metabolism. J Cell Biochem.

[49] Keyak JH, Koyama AK, LeBlanc A, Lu Y, Lang TF (2009). Reduction in proximal femoral strength due to long-duration spaceflight. Bone.

[50] Kawao N, Morita H, Nishida K, Obata K, Tatsumi K, Kaji H (2018). Effects of hypergravity on gene levels in anti-gravity muscle and bone through the vestibular system in mice. J Physiol Sci.

[51] Shimoide T, Kawao N, Tamura Y, Morita H, Kaji H (2016). Novel roles of FKBP5 in muscle alteration induced by gravity change in mice. Biochem Biophys Res Commun.

[52] Kawao N, Morita H, Obata K, Tatsumi K, Kaji H (2018). Role of follistatin in muscle and bone alterations induced by gravity change in mice. J Cell Physiol.

[53] Kawao N, Moritake A, Tatsumi K, Kaji H (2018). Roles of irisin in the linkage from muscle to bone during mechanical unloading in mice. Calcif Tissue Int.

[54] Heer M, Paloski WH (2006). Space motion sickness: incidence, etiology, and countermeasures. Auton Neurosci.

[55] Lintault LM, Zakrzewska EI, Maple RL, Baer LA, Casey TM, Ronca AE, Wade CE, Plaut K (2007). In a hypergravity environment neonatal survival is adversely affected by alterations in dam tissue metabolism rather than reduced food intake. J Appl Physiol.

[56] Morita H, Obata K, Abe C, Shiba D, Shirakawa M, Kudo T, Takahashi S (2015). Feasibility of a short-arm centrifuge for mouse hypergravity experiments. PLoS ONE.

[57] Morton GJ, Cummings DE, Baskin DG, Barsh GS, Schwartz MW (2006). Central nervous system control of food intake and body weight. Nature.

[58] Schwartz MW, Woods SC, Porte D, Seeley RJ, Baskin DG (2000). Central nervous system control of food intake. Nature.

[59] Balaban CD, Thayer JF (2001). Neurological bases for balance-anxiety links. J Anxiety Disord.

[60] Sonoda S, Yoshimura M, Abe C, Morita H, Ueno H, Motojima Y, Saito R, Maruyama T, Hashimoto H, Tanaka Y, Ueta Y (2018). Effects of hypergravity on the gene expression of the hypothalamic feeding-related neuropeptides in mice via vestibular inputs. Peptides.

[61] Itoi K, Seasholtz AF, Watson SJ (1998). Cellular and extracellular regulatory mechanisms of hypothalamic corticotropin-releasing hormone neurons. Endocr J.

[62] Vettor R, Fabris R, Pagano C, Federspil G (2002). Neuroendocrine regulation of eating behavior. J Endocrinol Invest.

[63] Abe C, Ueta Y (1985). Morita H (2013) Exposure to hypergravity during the preweaning but not postweaning period reduces vestibular-related stress responses in rats. J Appl Physiol.

[64] Diano S, Horvath B, Urbanski HF, Sotonyi P, Horvath TL (2003). Fasting activates the nonhuman primate hypocretin (orexin) system and its postsynaptic targets. Endocrinology.

[65] Yoshimura M, Matsuura T, Ohkubo J, Ohno M, Maruyama T, Ishikura T, Hashimoto H, Kakuma T, Yoshimatsu H, Terawaki K, Uezono Y, Ueta Y (2013). The gene expression of the hypothalamic feeding-regulating peptides in cisplatin-induced anorexic rats. Peptides.

[66] Hammam E, Dawood T, Macefield VG (2012). Low-frequency galvanic vestibular stimulation evokes two peaks of modulation in skin sympathetic nerve activity. Exp Brain Res.

[67] Hammam E, Macefield VG (2017). Vestibular modulation of sympathetic nerve activity to muscle and skin in humans. Front Neurol.

[68] Vinkers CH, Groenink L, van Bogaert MJ, Westphal KG, Kalkman CJ, van Oorschot R, Oosting RS, Olivier B, Korte SM (2009). Stress-induced hyperthermia and infection-induced fever: two of a kind?. Physiol Behav.

[69] Monson CB, Oyama J (1984). Core temperature of tailless rats exposed to centrifugation. Physiologist.

[70] Monson CB, Patterson SL, Horowitz JM (1985). Oyama J (1989) Thermoregulation in hypergravity-acclimated rats. J Appl Physiol.

[71] Oka T (2018). Stress-induced hyperthermia and hypothermia. Handb Clin Neurol.

[72] Sunagawa GA, Takahashi M (2016). Hypometabolism during daily torpor in mice is dominated by reduction in the sensitivity of the thermoregulatory system. Sci Rep.

[73] Gordon CJ (2001). The therapeutic potential of regulated hypothermia. Emerg Med J.

[74] Tu L, Poppi L, Rudd J, Cresswell ET, Smith DW, Brichta A, Nalivaiko E (2017). Alpha-9 nicotinic acetylcholine receptors mediate hypothermic responses elicited by provocative motion in mice. Physiol Behav.

[75] Gordon CJ (1983). Influence of heating rate on control of heat loss from the tail in mice. Am J Physiol.

[76] Rand RP, Burton AC, Ing T (1965). The tail of the rat, in temperature regulation and acclimatization. Can J Physiol Pharmacol.

[77] Ngampramuan S, Cerri M, Del Vecchio F, Corrigan JJ, Kamphee A, Dragic AS, Rudd JA, Romanovsky AA, Nalivaiko E (2014). Thermoregulatory correlates of nausea in rats and musk shrews. Oncotarget.

[78] Gordon CJ (2017). The mouse thermoregulatory system: its impact on translating biomedical data to humans. Physiol Behav.

[79] Madden CJ, Santos da Conceicao EP, Morrison SF (2017). Vagal afferent activation decreases brown adipose tissue (BAT) sympathetic nerve activity and BAT thermogenesis. Temperature.

[80] Cuccurazzu B, Halberstadt AL (2008). Projections from the vestibular nuclei and nucleus prepositus hypoglossi to dorsal raphe nucleus in rats. Neurosci Lett.

[81] Balaban CD, Beryozkin G (1994). Vestibular nucleus projections to nucleus tractus solitarius and the dorsal motor nucleus of the vagus nerve: potential substrates for vestibulo-autonomic interactions. Exp Brain Res.

[82] Cai YL, Ma WL, Wang JQ, Li YQ, Li M (2008). Excitatory pathways from the vestibular nuclei to the NTS and the PBN and indirect vestibulo-cardiovascular pathway from the vestibular nuclei to the RVLM relayed by the NTS. Brain Res.

[83] Cui J, Iwase S, Mano T, Kitazawa H (1999). Responses of sympathetic outflow to skin during caloric stimulation in humans. Am J Physiol.

[84] Abe C, Iwata C, Shiina T, Shimizu Y, Morita H (2011). Effect of daily linear acceleration training on the hypergravity-induced vomiting response in house musk shrew (*Suncus murinus*). Neurosci Lett.

[85] Sato G, Uno A, Horii A, Umehara H, Kitamura Y, Sekine K, Tamura K, Fukui H, Takeda N (2009). Effects of hypergravity on histamine H1 receptor mRNA expression in hypothalamus and brainstem of rats: implications for development of motion sickness. Acta Otolaryngol.

[86] Uno A, Takeda N, Horii A, Morita M, Yamamoto Y, Yamatodani A, Kubo T (1997). Histamine release from the hypothalamus induced by gravity change in rats and space motion sickness. Physiol Behav.

[87] Nobel G, Tribukait A, Mekjavic IB, Eiken O (2012). Effects of motion sickness on thermoregulatory responses in a thermoneutral air environment. Eur J Appl Physiol.

[88] Nalivaiko E, Rudd JA, So RH (2014). Motion sickness, nausea and thermoregulation: the "toxic" hypothesis. Temperature.

[89] Liu E, Lewis K, Al-Saffar H, Krall CM, Singh A, Kulchitsky VA, Corrigan JJ, Simons CT, Petersen SR, Musteata FM, Bakshi CS, Romanovsky AA, Sellati TJ, Steiner AA (2012). Naturally occurring hypothermia is more advantageous than fever in severe forms of lipopolysaccharide- and *Escherichia coli*-induced systemic inflammation. Am J Physiol Regul Integr Comp Physiol.

[90] Romanovsky AA, Shido O, Sakurada S, Sugimoto N, Nagasaka T (1997). Endotoxin shock-associated hypothermia. How and why does it occur?. Ann N Y Acad Sci.

[91] Romanovsky AA, Szekely M (1998). Fever and hypothermia: two adaptive thermoregulatory responses to systemic inflammation. Med Hypotheses.

